# Belowground Productivity in Grasslands and Savannas Depends on Shifting Fire and Herbivory Regimes

**DOI:** 10.1111/gcb.71202

**Published:** 2026-11-13

**Authors:** Courtney M. Currier, Christian Parrinello, Charlotte Dick, David J. Augustine, John M. Blair, Elizabeth H. Boughton, Corli Coetsee, Aaron S. David, Justin D. Derner, Sarah E. Hobbie, Forest Isbell, Kamogelo S. Modimola, Peter B. Reich, Benjamin Wigley, Kally Worm, Lydia H. Zeglin, Adam Pellegrini

**Affiliations:** ^1^ Department of Plant Sciences University of Cambridge Cambridge UK; ^2^ Department of Earth System Science, Doerr School of Sustainability Stanford University Stanford California USA; ^3^ Rangeland Resources & Systems Research Unit, USDA‐Agricultural Research Service Center for Agricultural Resources Research Fort Collins Colorado USA; ^4^ Division of Biology Kansas State University Manhattan Kansas USA; ^5^ Archbold Biological Station and Buck Island Ranch Lake Placid Florida USA; ^6^ Savanna Research Node, Scientific Services, SANParks Skukuza South Africa; ^7^ School of Natural Resource Management Nelson Mandela University, George Campus George South Africa; ^8^ Archbold Biological Station Venus Florida USA; ^9^ Department of Ecology, Evolution and Behavior University of Minnesota St. Paul Minnesota USA; ^10^ Environmental and Geographical Science University of Cape Town Rondebosch South Africa; ^11^ Department of Forest Resources University of Minnesota St. Paul Minnesota USA; ^12^ Institute for Global Change Biology, School for the Environment and Sustainability University of Michigan Ann Arbor Michigan USA; ^13^ Plant Ecology University of Bayreuth Bayreuth Germany

**Keywords:** aboveground productivity, belowground productivity, disturbance, fire, herbivory

## Abstract

In grasslands and savannas, most plant biomass and net primary productivity is belowground. How shifts in disturbance regimes, such as fire or large herbivore activity, impact belowground net primary productivity (BNPP) is unclear across climate‐driven productivity gradients. We tested the hypothesis that altered fire and herbivory regimes impact BNPP by changing the relative biomass of herbaceous and woody plants, which differ in their relative allocation to BNPP, and that climate constrains how much disturbance shifts this herbaceous–woody balance. We measured BNPP and aboveground vegetation across seven African and North American grasslands and savannas, with three long‐term fire manipulation levels (high frequency, intermediate frequency, and suppressed) and, at six sites, two long‐term herbivory levels (large herbivores excluded or open to large herbivores). Sites spanned an aridity index (mean annual precipitation [mm]/potential evapotranspiration [mm]) gradient—and associated woody thickening potential—from 0.21 to 0.71. BNPP increased with water availability. Fire and herbivory increased BNPP, with greater effects in wetter sites. In the wettest sites, BNPP gains under disturbance correlated with herbaceous biomass gains, as herbivory and frequent fire reduced woody plant cover relative to disturbance exclusion plots. BNPP responses were also predicted by root biomass, explaining why wetter sites and frequently disturbed plots within sites had higher BNPP. Climate determined how much the positive response of root biomass offset fire and grazing reductions in aboveground plant stocks, largely because water availability constrained the total aboveground plant biomass achieved without disturbance. Therefore, disturbances stimulated BNPP in wet savannas, largely by limiting woody thickening and promoting herbaceous growth with higher BNPP rates, whereas BNPP in drier and mesic sites tended to be resilient. Our findings show that belowground processes in grasslands and savannas fundamentally depend on disturbance regimes, climate‐driven productivity gradients, and their interactions, with important implications for understanding global carbon storage.

## Graphical abstract

Long‐term experiments across a climate gradient show belowground net primary productivity (BNPP) in grasslands and savannas depends on fire and herbivory. Disturbance stimulated BNPP in wet savannas by limiting woody thickening and promoting herbaceous growth while drier sites remained resilient, revealing climate‐disturbance effects belowground have major implications for carbon storage.

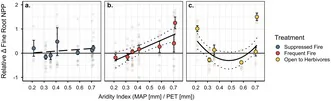

## 1 Introduction

Roots play a crucial role in the cycling of carbon (C) in terrestrial ecosystems (Jackson et al. 1997; McCormack et al. 2015; Norby and Jackson 2000). Soil organic C is highly correlated with root abundance across biomes (Jobbágy and Jackson 2000; Souza et al. 2023), and belowground inputs are the most efficient pathway for soil organic C formation (Sokol and Bradford 2019). In grasslands and savannas, which together account for ca. 45% of global net primary productivity (NPP) (Prăvălie 2016), the dominant C pool is belowground as root biomass and soil organic matter (Eswaran et al. 1993), and globally this pool stores as much C as the live biomass of forests (Pan et al. 2011). Productivity in grasslands and savannas is primarily constrained by water availability, but these systems are also shaped by fire and large herbivores and account for approximately 80% of the global mean annual burned area (Giglio et al. 2018) and 50% of land managed for grazing (Asner et al. 2004; Kelly et al. 2020). Yet, human activity is drastically altering fire and herbivory regimes globally (Andela et al. 2017; Asner et al. 2004). Therefore, understanding the impact of altered disturbance regimes on belowground stocks and belowground net primary productivity (BNPP) in grasslands and savannas is ultimately important to understanding terrestrial C cycling globally.

Across grasslands and savannas, NPP generally increases with mean annual precipitation (MAP), with the vast majority of studies focusing on aboveground net primary productivity (ANPP) (Cleveland et al. 2011; Huxman et al. 2004; Knapp et al. 2017; Knapp and Smith 2001; Lauenroth and Sala 1992; Ponce‐Campos et al. 2013; Sala et al. 2012; Schuur 2003; Yang et al. 2008). In addition to water availability, grasslands and savannas are also strongly shaped by fire (Bond and Keeley 2005; Kelly et al. 2023; Pausas et al. 2025; Pausas and Ribeiro 2013) and large herbivores (Oesterheld et al. 1999; Pringle et al. 2023). Disentangling the controls on grassland and savanna productivity is therefore complex, as water availability versus disturbance hypotheses compete in explaining tree–grass coexistence and their joint contributions to productivity. Recent models explain relative dominance by grasses or trees by ecohydrological constraints in dry systems and disturbance controls in wet savannas (Holdo and Nippert 2023). Therefore, the consequences of altered disturbance regimes in the Anthropocene on grassland and savanna productivity are not spatially uniform, depending on water availability and the degree to which woody thickening is affected (Oesterheld et al. 1999; Reich et al. 2001).

Two mechanisms could explain BNPP patterns under altered disturbance regimes. First, both woody and herbaceous plants contribute fine roots to the total root pool, but the relative contribution is not necessarily equal. For example, in a temperate savanna, BNPP diverged with differences in tree versus grass dominance even under similar fire frequency, and while grass‐dominated plots were associated with higher root stocks, tree‐dominated stands had higher BNPP (Reich et al. 2001). The relative fraction of BNPP attributed to trees may therefore scale with increasing water availability and the ability for wet savannas to support higher density of trees under fire exclusion. Wet savannas with the ability to support higher tree density under disturbance exclusion may therefore exhibit larger responses to altered disturbance. While fire consumes both woody and herbaceous plants (Higgins et al. 2007), herbivory may be more selective depending on animal community assemblages and plant phenology. Whether reduction of herbivore activity impacts trees or herbaceous plants more depends on whether dominant herbivores are browsers (consuming woody plants), grazers (consuming herbaceous plants), or mixed feeders (consuming both) (Pellegrini et al. 2017) as well as forage availability (Abraham et al. 2019).

The second mechanism underlying BNPP is changes to plant allocation strategies under disturbance. Plants may increase investment belowground under disturbance exclusion for competitive advantage or under frequent disturbance to optimize resource acquisition for re‐growth. Optimizing root growth for water acquisition in different soil layers (c.f., Walter's two‐layer hypothesis (Walter 1939)) is one important mechanism explaining tree–grass coexistence (Holdo and Nippert 2023). Vegetation in dry sites may already have relatively higher investment in deep roots for water uptake and thus changes to BNPP are a relatively small investment if the structures already exist. Increases in disturbance result in losses of soil nutrients, especially in the case of long‐term directional increases in fire frequency (Johnson and Matchett 2001; Pellegrini et al. 2018), thereby stimulating fine root production for acquisition of nutrients. Increases in disturbance frequency could also stimulate BNPP due to re‐sprouting or compensatory growth strategies (Knapp et al. 2012; Menges et al. 2020; Oesterheld and McNaughton 1991) that optimize acquisition of temporally‐explicit increases in light availability or localized nutrient hotspots excreted by herbivores (López‐Mársico et al. 2015).

Many past studies investigated aboveground (Briggs and Knapp 1995; Buis et al. 2009; Heisler et al. 2004; Oesterheld et al. 1999) and belowground (Johnson and Matchett 2001; Milchunas and Lauenroth 1993; Pellegrini et al. 2020; Sims and Singh 1978) responses to fire and herbivory, sometimes jointly and with many individual sites having strong long‐term histories monitoring these responses (Biondini et al. 1998; Brown and Collins 2023; Reich et al. 2001). However, despite growing cross‐site BNPP datasets (e.g., Sun et al. 2023) and syntheses concerning other global change drivers (e.g., Wilcox et al. 2017; Yaffar et al. 2024), no study has systematically considered how altered disturbance regimes underly BNPP across a large climate gradient of grassland‐savannas.

To address this research gap, we asked four questions: (1) How does BNPP in grasslands and savannas change across a broad climate gradient? (2) How does climate modify effects of fire and herbivory on BNPP? (3) What mechanisms underlie shifts in BNPP? (4) How do shifts in BNPP impact total biomass C storage? We hypothesized that: (1) BNPP increases with water availability; (2) water availability modulates the effects of fire and herbivory on BNPP; (3) these responses can be explained by shifts in woody versus herbaceous dominance and/or belowground allocation; and (4) disturbance‐induced increases in BNPP/root stocks compensate, at least partially, for losses in aboveground biomass.

Here, we examined how climate interacts with fire and herbivory, separately, to shape BNPP using long‐term field manipulations at seven sites on two continents. We used aridity index (UNEP AI = mean annual precipitation/mean annual potential evapotranspiration; Middleton and Thomas 1992; Zomer et al. 2022) to frame our results in a climate context, as sites spanned a range of 0.21 (semi‐arid) to 0.71 (humid). We do not use aridity as a space‐for‐time proxy to infer plant responses to drought (c.f., Knapp et al. 2026), but rather to frame plant responses to disturbance as vegetation assemblages and plant traits along aridity gradients reflect evolutionary histories and adaptations to long‐term relative water availability. We collected 376 aboveground biomass samples paired with belowground root standing stocks and root in‐growth cores to assess BNPP down to 20 cm in 64 experimental plots distributed across the long‐term fire and herbivory manipulations. We focus on fine root NPP for this study, as fine roots are important mediators for soil C cycling compared to coarse and woody roots (Gill and Jackson 2000; McCormack et al. 2015). We also quantified woody layer density within experimental plots. The study sites span a broad range of climate, vegetation structure, and large herbivore types typical of grasslands and savannas across North America and South Africa.

## 2 Materials and Methods

### 2.1 Overview of Study Sites

We selected seven sites with long‐term fire and herbivory manipulation experiments (Figure S1) across a range of climates, soil types, large herbivore assemblages, and dominant vegetation (Table S1). In order of aridity, they are: Central Plains Experimental Range (CPER; CO, USA), Satara Experimental Burn Plots and University of Cape Town Exclosure (SAT; Kruger National Park, South Africa), Skukuza Experimental Burn Plots and N'Kuhlu Exclosure (SKU; Kruger, South Africa), Pretoriuskop Experimental Burn Plots (PKOP; Kruger, South Africa), Konza Prairie Biological Station (KPBS; KS, USA), Archbold Biological Station (ABS; FL, USA), and Cedar Creek Long Term Ecological Research Site (CDR; MN, USA). Each site represents a characteristic savanna or grassland ecosystem for that region, where one or both disturbance regimes have historically shaped the landscape vegetation dynamics. A detailed description of all study sites is included in the Supporting Information. Long‐term fire and herbivore manipulations are detailed in Table S1. Sample collection took place during the October 2023–April 2024 growing season for Kruger sites and April/May–September/October 2024 growing season for North American sites. Long‐term annual and growing season rainfall averages were reported using multi‐decadal weather data from each site. The study year growing season precipitation amount fell within or close to the historical range for 6 out of 7 sites, with CDR receiving 28% less rainfall than usual during the growing season (Figure S1, table inset). Long‐term aridity index values were extracted over the years 1970–2000 for each site from the Global Aridity and PET Database (Zomer et al. 2022) using the terra package in R (Hijmans et al. 2023).

### 2.2 Experimental Design

Fire treatments included three levels at each study site: unburned or long‐term fire suppression, an intermediate fire frequency typical of the historical regime for each site, and high fire frequency. In the African sites (Kruger), the fire treatments were all open to large native herbivores including grazers, browsers, and mixed feeders, but in North American sites, the experimental treatments used to compare fire effects alone excluded domestic grazers (cattle or bison), but not mixed feeders (e.g., deer, antelope) or other mammalian or invertebrate herbivores. Although white‐tailed deer, mule deer, and pronghorn exist at some of the North American sites, we did not consider the effects of these ungulates due to their smaller body size and lower forage consumed compared to stocked cattle and bison (c.f., Schoenecker et al. 2022).

Herbivory manipulations in Kruger consisted of long‐term exclosures exposed to natural fire ignition but otherwise fire was suppressed (e.g., not a part of an experimental fire manipulation). This experimental choice is critical for understanding the role of browsers on large trees in our study, where elephant ( *Loxodonta africana* ) activity, for example, is known to affect woody plant dynamics more than fire alone and elephants remove increasing amounts of woody biomass across a Kruger productivity gradient (Pellegrini et al. 2017).

Grazed plots in North American sites were paired with ungrazed plots under the same fire frequency treatment (either intermediate or frequent) typical of land management strategies for each site, resulting in grazed‐burned versus ungrazed‐burned comparisons. For example, because frequent prescribed burning (every 1*–*2 years) is generally used in Kansas and southcentral Florida to promote forage for grazing, the grazed grasslands at KPBS and ABS were paired with ungrazed plots under the same frequent‐fire regime. This choice reflects scenarios where grazing activity and fire would similarly have the largest effects on herbaceous layer productivity in these sites, as cattle or bison would typically avoid forested patches in unburned plots where herbaceous forage is less available or altogether absent. The exception was CPER, where the fire experiment was conducted in a pasture where cattle were excluded, and effects of herbivory were evaluated by sampling inside and outside exclosures within two pastures grazed at a moderate cattle stocking rate, and one pasture at a heavy cattle stocking rate.

Up to three experimental replicates (*n* = 3) for each of three categorical treatments (fire treatments while holding grazing constant: suppressed fire, intermediate fire, frequent fire; herbivore treatments while holding fire constant: large herbivores excluded, open to large herbivores) were selected for sampling when possible (fire: *N* = 9 per site; herbivory: *N* = 2 per site, except PKOP). Because prescribed burning at some sites occurs at large, watershed scales, three experimental replicates were not always possible, as indicated in Table S1.

### 2.3 Vegetation Biomass Sampling

Within each experimental plot, we selected five sub‐plot pseudoreplicates (hereafter “sampling points”) within a 50 × 75 m area (10–15 sampling points per treatment, *N* = 45–60 total per site). These sampling points were randomly generated using QGIS after excluding a 25 m buffer around the experimental plot edges. Sampling points were relocated in situ if they fell on a topographic or ecological anomaly, such as a termite mound. Pseudoreplication was handled statistically and described in the Analyses section below.

To measure belowground stocks, one standing root core was taken to 20 cm depth at each sampling point. To measure belowground productivity (BNPP), root in‐growth cores were installed at the beginning of the growing season (late spring) at each sampling point and recovered during peak herbaceous plant biomass of the same year (approximately early autumn for each site, ~3 months after installation). The in‐growth cores were constructed of polypropylene mesh with 2 mm openings, 20 cm in depth, and 5 cm in diameter and filled with root‐free soil. All roots were picked from soil using forceps and sorted into diameter size classes of coarse (≥ 2 mm) or fine (< 2 mm). Roots were gently washed to remove any soil or particulates and dried at 60°C for 48 h before weighing. Standing root stock was expressed as g m^−2^, and root productivity was calculated as the in‐growth core root stock divided by the number of days left in the field scaled to an annual basis (g m^−2^ year^−1^). We assumed that the majority of root growth, primarily for fine roots targeted by our method, occurs during the growing season as the 2 mm mesh may have under‐sampled coarse roots. Root turnover was then calculated as the root production divided by the standing root stock (year^−1^).

To measure standing herbaceous layer aboveground biomass, we clipped a 20 × 50 cm area from the herbaceous layer during peak biomass at each sampling point and sorted the aboveground plant biomass according to general functional type: forbs, sub‐shrubs or seedlings, and graminoids. Within each functional type, biomass was further sorted by color to assess living status: gray (standing dead), yellow (senesced), and green (alive). Biomass was dried at 60°C for 48 h and then weighed to give a standing stock in g m^−2^. We present living (green) stock. Root mass fraction was calculated as the proportion of fine root biomass to total herbaceous layer living biomass.

Overstory woody plant stem density and biomass were estimated at the plot‐level (not pseudoreplicated) for each site. Depending on woody species density and the feasibility of counting and estimating all individuals within a site, pre‐determined plots sized from 25 × 25 m up to 50 × 75 m in area were established in each experimental replicate. All woody stems with heights ≥ 0.5 m were counted within the sampling area to calculate stem density as individuals per m^2^. Woody biomass was then estimated within the pre‐determined sampling area using site‐specific allometric equations, and total stock of both wood and leaf components was calculated as g m^−2^.

### 2.4 Data Analyses and Statistics

Climate effects on fine root NPP, fine root stocks, fine root turnover, aboveground woody stem density, and herbaceous biomass were assessed using generalized linear mixed‐effects models implemented with the glmmTMB package (Bolker 2019; Brooks et al. 2017) in R (Team 2013). To account for meaningful zeros in biomass, models used a Tweedie error distribution with a log link function. Aridity index was included as a fixed effect, while plot nested within site was specified as a random effect for all response variables measured at the sub‐plot level except woody stem density, which was measured at the plot level (and thus site was the only random effect). Alternative model forms (e.g., linear vs. polynomial relationships with aridity) were compared using Akaike Information Criterion (AIC) (Sakamoto et al. 1986), and models within ΔAIC ≤ 2 were considered equally supported, with the most parsimonious model selected as the final model. Model predictions were then back‐transformed for visualization.

To assess the effects of climate and disturbance on fine root NPP, fine root NPP was first relativized across all sites using reference treatments for fire and herbivory manipulations. Relative change in fine root NPP was calculated by scaling individual treatment observations relative to the mean value of the reference treatment across plots, where intermediate fire frequency and herbivore exclosures were selected as the reference treatments (Table S1; e.g., (annual burning − triennial burning)/triennial burning, respectively, in the Kruger sites):

$$\begin{array}{c} \text{Relative ∆ fine root}\;\mathrm{NPP}=\\ \left(\text{treatment}–\text{mean}\;\left[\text{reference}\right]\right)/\text{mean}\;\left(\text{reference}\right) \end{array}$$

This approach retained plot‐level variation of the response—which was subsequently addressed in mixed effects modeling approach described below—relative to a common reference condition. Using relative change also allowed disturbance responses to be compared across sites with different baseline productivity levels.

As a robustness check, we also calculated the Relative Interaction Index (Armas et al. 2004), which provides a symmetric alternative to the log response ratio and can handle biologically meaningful zeros:

$$\begin{array}{c} \text{Relative interaction index}=\\ \left(\text{treatment}–\text{mean}\;\left[\text{reference}\right]\right)/\left(\text{treatment}+\text{mean}\;\left[\text{reference}\right]\right) \end{array}$$

Again, we used the mean of reference treatments to produce scaling constants for each observation in treatment plots (c.f., Ohlert et al. 2026) and accounted for plot‐level variability using mixed effects models. Because both approaches produced similar results, relative response values were used in the main analyses for ease of interpretation. Thus, we present relative responses in the main text for interpretability, but statistical outcomes using RII values are reported alongside statistics using relative values in the Supplemental Material. Relative changes in fine root NPP were then analyzed using generalized mixed effects models for each disturbance treatment with aridity index as the fixed effect and plot as the random effect; variance associated with site was close to 0 for all models except fine root NPP under frequent fire, and thus site was omitted from model random effects. Furthermore, a Tweedie distribution was not required for these models and a Gaussian distribution was used. All model assumptions were assessed using standard diagnostics, including quantile‐quantile plots and inspection of residual distributions.

Relationships between fine root NPP with aboveground (living woody stems, living herbaceous layer stocks) and belowground (fine root stock, fine root turnover) predictors were tested via regressions. Models were again fitted using generalized linear mixed‐effects models in glmmTMB within R, using a Tweedie error distribution with a log link function and fit separately for each disturbance treatment. The exception was the Suppressed Fire treatment model, which failed to converge under the Tweedie distribution; in this case, a Gamma distribution with a log link was used instead. Because the Gamma distribution cannot accommodate zero values, but the Suppressed Fire subset contained no zero values of fine root NPP, this substitution was appropriate and functionally equivalent. The aboveground models were assessed using a multivariate approach to compare the relative strength of z‐score standardized living herbaceous layer stocks versus stem densities on fine root NPP responses, with plot as the random effect. Belowground explanatory models were fitted using univariate predictors of fine root stock and fine root turnover, and thus left unscaled, with plot as the random effect. A separate model was run for each disturbance treatment, and best‐fit models comparing linear or polynomial fixed effects were again selected using AIC. When singular fits occurred, the nested random effect of site was removed. Model predictions were then back‐transformed for visualization.

Finally, to estimate system‐level changes in plant biomass pools, we calculated total stocks by computing the relative change in woody biomass, herbaceous biomass, and fine root stocks to reference treatments. Relative net ecosystem change was then calculated using the weighted relative change of each pool to the total biomass pool.

## 3 Results

### 3.1 Fine Root Stocks and BNPP Increased With Water Availability

Fine root NPP varied by 4‐fold across the gradient and increased with water availability, ranging from 158 ± 13 to 636 ± 84 g m^−2^ year^−1^ (mean ± standard error) (Figure 1a; Table S2). Fine root stocks similarly increased with water availability, rising from 150 ± 13 to 1432 ± 133 g m^−2^ (Figure 1b; Table S2). In contrast, fine root turnover had a quadratic relationship with water availability, with the driest and wettest sites having the lowest values (0.44 +/‐ 0.048 and 0.30 +/‐ 0.056  year^−1^, respectively) and sites with intermediate aridity having the highest values, peaking at 1.73 ± 0.22 year^−1^ (Figure S2; Table S2). This translates to mean residence times (1/turnover) of approximately 2.3 to 3.3 years at the driest and wettest ends of our aridity gradient, respectively, and 0.624% years at intermediate aridity.

**FIGURE 1:**
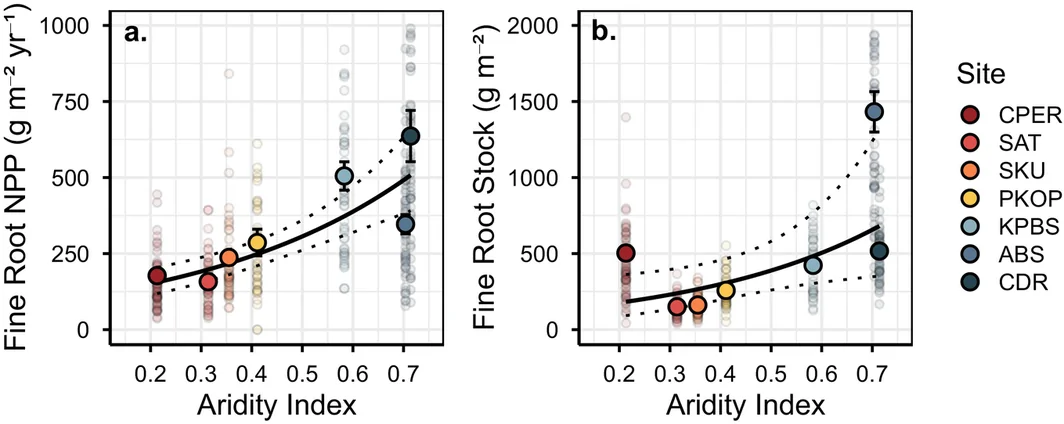
Belowground relationships of (a) fine root NPP (g m^−2^ year^−1^) and (b) fine root stocks (g m^−2^ year^−1^) with climate (UNEP aridity index as mean annual precipitation [mm]/potential evapotranspiration [mm]). Large points represent site‐level means, regardless of disturbance treatment, ± standard error. Light points represent raw values, and solid lines indicate significant predicted fits back‐transformed from generalized mixed effects models using Tweedie distribution and log‐link, with plot nested within site as the random effect for response variables. Dotted lines represent 95% confidence intervals around predicted fit. *N* = 752 root cores, (376 standing stock and 376 in‐growth). Statistical output is reported in Table S2.

Total root NPP across sites was almost entirely comprised of fine root NPP, with a range of mean contributions from 93% ± 8% to 100% across sites (Table S3). Although coarse roots in the top 20 cm comprised up to approximately half of the standing stock, depending on site and disturbance treatment, their turnover was very slow (often close to 0), indicating low coarse root turnover that was not detectable in our root in‐growth core sampling interval, which was a few months across the growing season. Across all sites, fine roots comprised 57% ± 4% to 97% ± 2% of the standing stocks in the top 20 cm (aggregating across treatments). We note that fine root stocks were significantly greater than expected based on the aridity index at two sites on opposite ends of the aridity gradient (CPER and ABS), which we suggest is due to species‐specific root traits of dominant plant species at each site. CPER is dominated by a prostrate, grazing‐tolerant perennial grass ( *Bouteloua gracilis* ) that maintains high belowground root reserves (Milchunas 2009). The plant community at ABS includes palmetto ( *Serenoa repens* and *Sabal etonia* ). We did not separate roots according to species, but palmetto has a higher belowground biomass allocation compared to other species at ABS (Saha et al. 2010), are coarser and heavier than a typical herbaceous fine root fraction separated with our method (< 2 mm diameter), and were visibly abundant in our samples due to their characteristic reddish‐brown color, white rings along root lengths, and lack of root hairs (Fisher and Jayachandran 1999).

### 3.2 Fire and Herbivory Effects on Fine Root NPP Depend on Site and Aridity

Fine root NPP responses to altered fire frequency (i.e., fire exclusion or more frequent fires) and herbivore presence/absence differed among sites (Figure 2). Fire exclusion had variable effects on fine root NPP: the maximum observed relative loss occurred at SAT, with −15% ± 9% under suppressed fire relative to intermediate fire, while the maximum observed relative gain occurred at PKOP, with +48% ± 57%. Frequent fire had relatively larger effects on BNPP than fire exclusion. With higher fire frequency, some sites showed declines in fine root NPP, with the maximum observed relative loss in fine root NPP occurred at CPER, with −18% ± 14% under frequent fire relative to intermediate fire; however, other sites showed increases in fine root NPP, with the maximum observed relative gain in fine root NPP at CDR, with +125% ± 24% under frequent fire relative to intermediate fire. Similarly, sites differed in fine root NPP in response to herbivory, with some showing increases and others decreases. The maximum observed relative loss in fine root NPP occurred at KPBS with −38% ± 4%, and the maximum observed relative gain in fine root NPP occurred at CDR with +150% ± 16%, both comparing NPP in plots with herbivores present relative to plots without herbivores.

**FIGURE 2:**
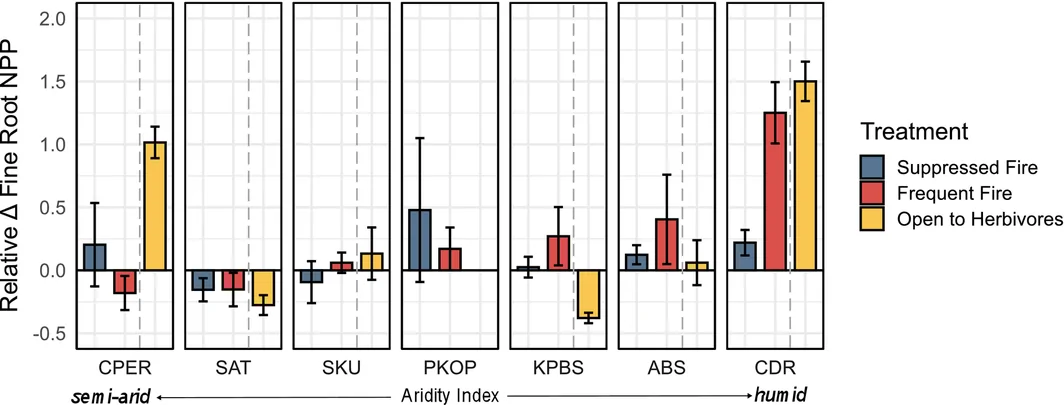
Relative change in fine root NPP within study sites. Relative change is defined as the value in the treatment relative to the “control”: Suppressed is (fire exclusion − intermediate)/intermediate, frequent is (increased frequency − intermediate)/intermediate, and open to herbivores is (herbivory − exclosure)/exclosure. Bars represent means ± standard error across plots within a treatment in a site. Site legend (in order from most arid to least arid): Central Plains Experimental Range (CPER), Satara (SAT), Skukuza (SKU), Pretoriuskop (PKOP), Konza Prairie Biological Station (KPBS), Archbold Biological Station (ABS), Cedar Creek LTER (CDR).

To test if climate modified the BNPP versus fire and herbivory responses from Figure 2, we regressed relative change in fine root NPP (per disturbance) against aridity index of each site. The resulting relationships were significant for frequent fire and herbivory (Figure 3, Table S4), but the shape of the correlation depended on the disturbance type. Increased fire frequency had smaller, negative effects in drier sites compared to larger, positive effects on relative fine root NPP in the wetter sites, increasing from −18% ± 14% in the driest site (CPER) to +125% ± 24% in the wettest site (CDR). In contrast to effects of increased fire frequency, the effects of fire suppression relative to baseline were not significantly related to aridity. Herbivore effects were quadratic, such that the largest, positive effects were in the driest (CPER) and wettest (CDR) sites, with +101% ± 13% and +150% ± 16% relative increases in fine root NPP, respectively.

**FIGURE 3:**
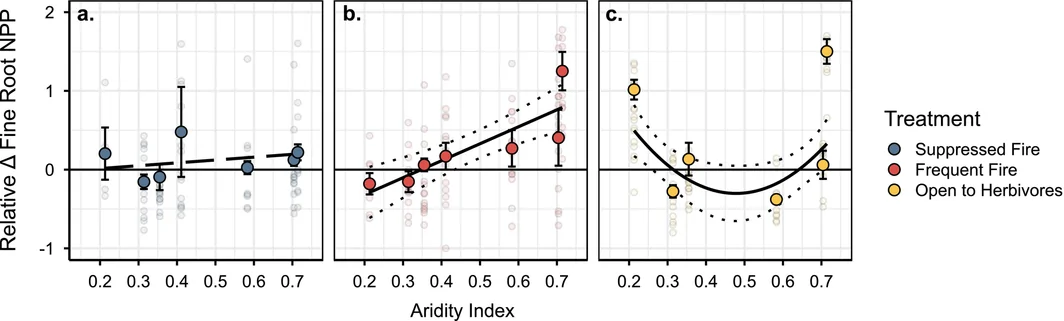
Relative change in fine root NPP across an aridity gradient under altered disturbance regimes, relative to reference treatments. Large points represent site‐level means for each disturbance treatment ± standard error, where (a) blue = suppressed fire relative to intermediate fire, (b) red = frequent fire relative to intermediate fire, and (c) yellow = open to large herbivores relative to herbivore exclusion. Solid lines indicate significant predicted fits from mixed effects models fitting relationships, with plot as the random effect. Statistical outputs are in Table S4.

### 3.3 Woody and Herbaceous Plant Abundance Together Explain BNPP Response to Disturbance

The differences in how disturbances affected fine root NPP can be explained by the relative changes in aboveground woody versus herbaceous plant abundance. The relationship between woody stem density and fine root NPP was significantly positive across all fire frequencies (Figure 4a; Table S5). In contrast, the relationship between herbaceous aboveground biomass and fine root NPP was only significantly positive in intermediate and frequently burned plots (Figure 4b; Table S5). The effect size of aboveground herbaceous biomass stock contribution to BNPP was stronger than the effect of woody stem density in intermediate burned plots (Table S5). The relative abundance of woody‐ versus herbaceous plants, and subsequent effects on BNPP, was explained by climate. Wetter sites had higher woody stem density, which was negatively associated with herbaceous biomass regardless of treatment (e.g., site‐level means) and disturbance exclusion only (Figures S3 and S4).

**FIGURE 4:**
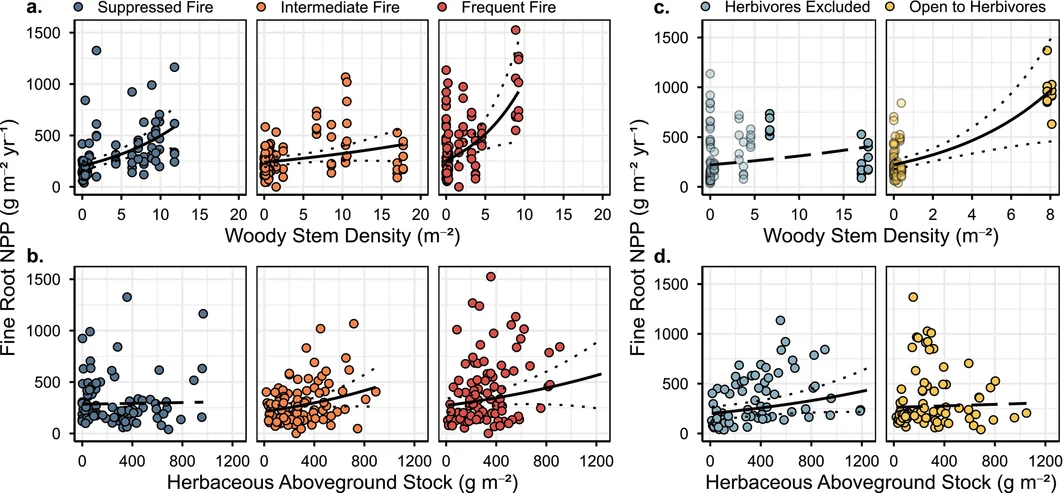
Fine root NPP (g m^−2^ year^−1^) versus aboveground vegetation explanatory variables (woody stem density [individuals/m^−2^] and living herbaceous layer stocks [g m^−2^ year^−1^]) under altered fire, (a, b) and herbivory, (c, d). Points represent sub‐plot values for each disturbance treatment. Dark points in (c) represent data from Cedar Creek (CDR). Solid lines indicate significant predicted fits ±95% confidence intervals (dotted lines) back‐transformed from multivariate, generalized mixed effects models using a Tweedie distribution and log‐link, with plot as the random effect. Statistical output is reported in Table S5.

Herbivore effects were similar to fire effects in terms of the relationship between woody stems and fine root NPP; there was a positive relationship between woody stem density and fine root NPP in plots that were open to herbivores (Figure 4c), but this pattern was driven by high stem density of oak saplings in our wettest site (CDR). Herbivore exclusion effects differed from fire exclusion effects; in plots where herbivores were excluded, living herbaceous layer stock and fine root NPP still exhibited a positive relationship (Figure 4d; Table S5). In conclusion, the abundance of both woody and herbaceous plants aboveground positively contributed to fine root NPP, with the relative importance associated with trees or herbaceous plants shifting with disturbance.

### 3.4 Shifts in Root Stocks, but Not Biomass Allocation, Also Explain Fine Root NPP

Fine root NPP was positively correlated with fine root stocks when all treatments were analyzed together (Figure S5; Table S2). Subsequent regressions revealed that fine root NPP was positively correlated with fine root stocks only in disturbed plots (frequent fire and herbivory; Figure 5; Table S5). Patterns in belowground biomass allocation did not explain changes in fine roots and NPP across sites or in response to disturbance treatments (Figure S6). Thus, productivity scaled with the total amount of fine root biomass present in the most disturbed plots, and changes in total fine root biomass were not a result of shifting relative biomass allocation.

**FIGURE 5:**
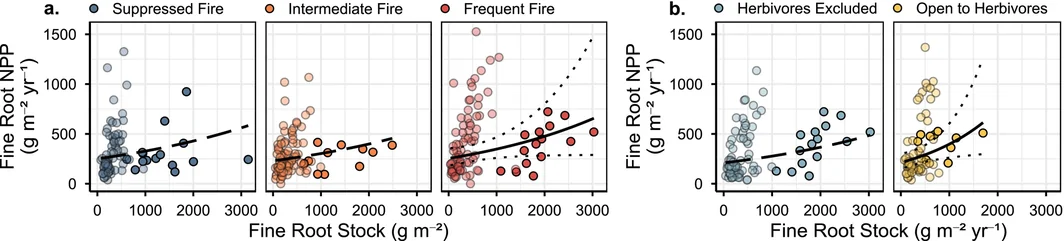
Fine root NPP (g m^−2^ year^−1^) versus fine root stock (g m^−2^) under altered disturbance regimes, (a) fire and (b) herbivory. Points represent sub‐plot values for each disturbance treatment. Solid lines indicate significant predicted fits ±95% confidence intervals (dotted lines) back‐transformed from generalized mixed effects models using a Tweedie distribution and log‐link, with plot as the random effect. Dark points represent data from Archbold Biological Station (ABS). Statistical output are in Table S5.

### 3.5 Roots Compensate for Changes Aboveground in Terms of Total Ecosystem Plant Biomass Stocks

We then assessed the relative change in above‐ and belowground plant biomass stocks and compared relative differences in the main biomass compartments (Figure 6). We present here the main differences between woody biomass, fine root biomass, and overall net change weighted by the stock size for woody aboveground, herbaceous aboveground, and fine root belowground biomass. The proportion of the net change in total biomass attributed to fine roots under each disturbance is described in Table S6.

**FIGURE 6:**
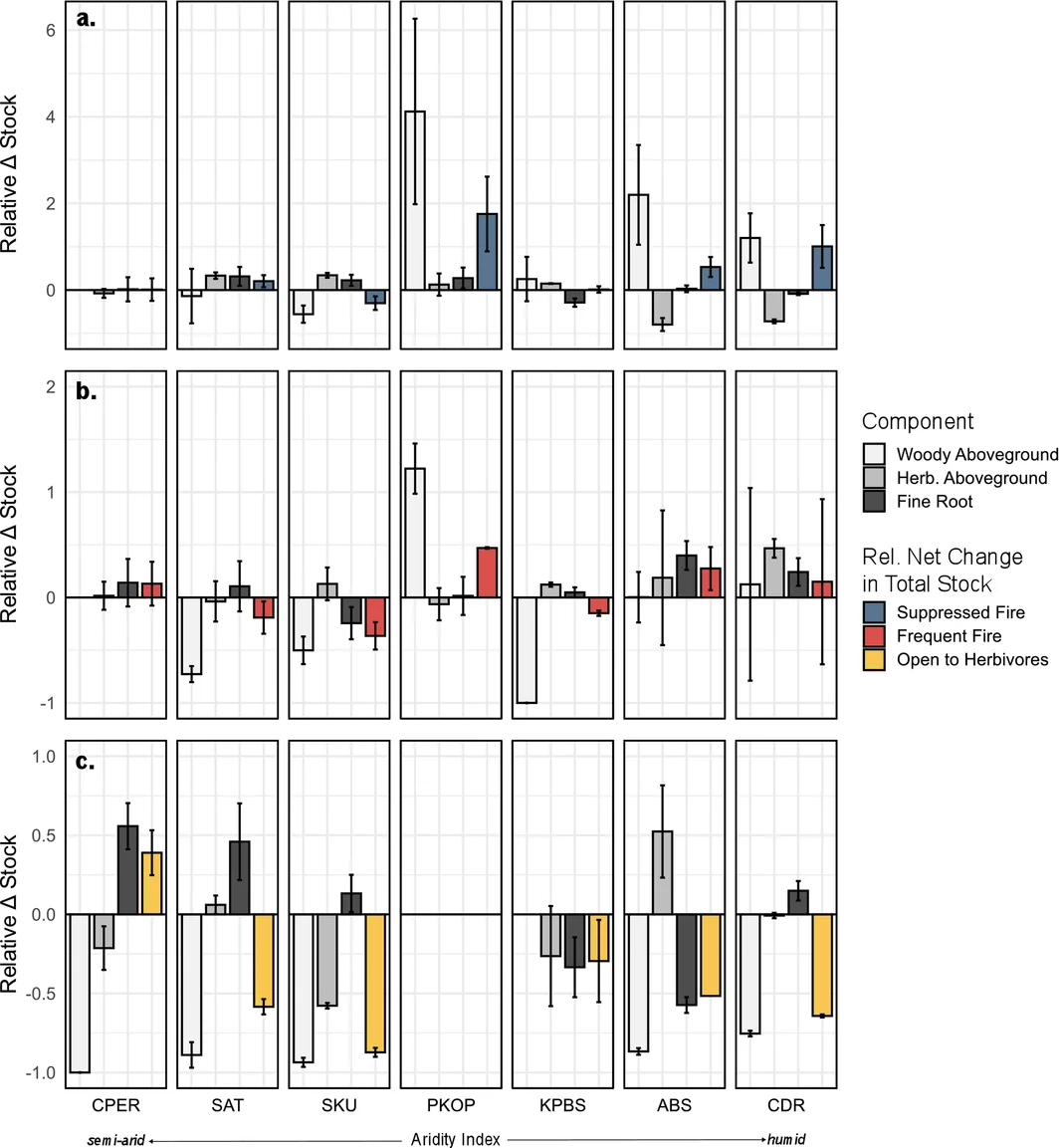
Relative change in stocks across an aridity gradient, parsed by partitioning to above‐ and belowground biomass and disturbance type: (a) suppressed fire, (b) frequent fire, (c) open to herbivores. Bars show the relative change in woody aboveground (white), herbaceous aboveground (light gray), and fine root (dark gray) biomass compared to treatment‐specific references (intermediate fire and herbivore exclusion). Blue, red, and yellow bars represent the weighted relative net change in total biomass stock (mean ± SE) for suppressed fire, frequent fire, and herbivory, respectively. Site legend (in order from most arid to least arid): Central Plains Experimental Range (CPER), Satara (SAT), Skukuza (SKU), Pretoriuskop (PKOP), Konza Prairie Biological Station (KPBS), Archbold Biological Station (ABS), Cedar Creek Ecosystem Science Reserve (CDR).

Under fire suppression, root biomass stocks did not always increase proportionally with gains in aboveground woody biomass (Figure 6a). The four wettest sites saw gains in woody biomass aboveground under fire suppression, but fine root biomass did not consistently increase. At PKOP, a +28% ± 24% root gain accompanied the much larger woody gain of +410% ± 214% under fire suppression, and there was a net gain in overall biomass of +176% ± 86%. At KPBS, a −29% ± 10% decline in roots offset the +25% ± 51% woody gain, reducing the net change to +1% ± 7%. At ABS, a +3% ± 8% gain in fine roots dampened the +220% ± 115% gain in woody stock, yielding a net stock gain of +53% ± 23%. At CDR, a −9% ± 3% decline in fine roots reduced the woody gain of +120% ± 57%, yielding a net gain of +101% ± 49%.

Under frequent fire (Figure 6b), any gains in root stocks often dampened or sometimes offset woody losses aboveground. For example, at SAT, a −73% ± 8% decline in woody stock was only modestly buffered by a +11% ± 24% gain in fine roots, yielding a net loss of −19% ± 15%. At KPBS, a +5% ± 5% gain in root stocks under frequent fire dampened the complete woody loss to an overall net loss of −15% ± 3%. At ABS, a +40% +/‐ 14% gain in root stocks contributed to a +28% +/‐ 20% gain in total stocks overall, up from +6% +/‐ 35% gain in woody biomass alone.

Finally, herbivory resulted in net losses of total biomass stocks across most sites (Figure 6c), except at the driest site (CPER), where near‐complete loss of woody stock was offset by a +56% ± 15% gain in fine roots, producing the only net gain observed under herbivory (+39% ± 14%). This result was driven by the presence of Artemisia shrubs in one grazing exclosure and the absence of woody plants outside exclosures. At SAT, a −89% ± 8% woody loss was buffered by a +46% ± 24% gain in fine roots, dampening the net loss to −58% ± 5%. At SKU, a −94% ± 3% woody loss was only modestly buffered by a +13% +/‐ 12%  root gain, yielding a net loss of −87% ± 3%.

Thus, compensatory responses in belowground biomass can be important for offsetting the losses of aboveground woody biomass under fire suppression, in some cases leading to gains in total plant biomass stocks despite frequent disturbance by fire, and only modestly offset losses aboveground due to herbivory.

## 4 Discussion

Our results demonstrate that belowground productivity in grasslands and savannas depends on site aridity, shifting fire and herbivory regimes, and their interactions. First, we supported our first hypothesis that fine root NPP was higher in wetter savannas, independent of disturbance effects. Second, water availability regulated the site‐level responses of fine root NPP to disturbances. Third, we identified that the degree to which woody plants shade herbaceous vegetation and shifts in woody and herbaceous dominance jointly influence fine root NPP across a disturbance–climate continuum. BNPP was not explained by any change in allocation between above and belowground biomass, but BNPP scaled with absolute change in root stocks. Lastly, we provided evidence for the compensatory role of shifts in root biomass in dampening or offsetting disturbance‐driven losses aboveground. Below, we discuss fire and herbivory effects in the context of this climate gradient, detail mechanistic explanations unique to specific sites that likely contributed to variability, and describe implications for C storage under global change.

### 4.1 Climate Driven Variability in NPP

Responses of BNPP to altered fire regimes differed across the aridity gradient, with the clearest aridity‐driven trend occurring under frequent fire. While we found little evidence for changes in fine root NPP with altered fire frequency in the drier grassland sites, increased fire frequency enhanced fine root NPP in the wetter savanna sites, with the magnitude of response increasing along the water availability gradient. Water or nutrient limitation might constrain recovery post‐fire in dry sites (Grünzweig et al. 2022), potentially indicated by the marginal relative losses in fine root NPP at CPER and SAT under frequent fire; but this decrease in BNPP did not translate to relative decreases in root stocks at these sites, indicating resiliency to disturbance. Increases in BNPP to disturbance in the mesic and wet sites conversely likely reflect optimized resource acquisition post‐disturbance by grass species in competition with forbs or woody plants. For example, past studies at KPBS showed frequent burning maintained dominance by the perennial C4 grass species *Andropogon gerardii* and increased BNPP over forb or shrub establishment (Kitchen et al. 2009), in congruence with our results. We therefore conclude from the multivariate analyses that maintaining an herbaceous layer contributes to high fine root NPP, and this was determined by fire and higher water availability. Woody stem density rather than biomass was selected to capture post‐disturbance dynamics in woody vegetation that may explain variation in fine root NPP, such as resprouting following fire, which would not be well‐represented by biomass estimates derived from site‐specific allometric equations with larger diameter at breast height cutoffs.

Grazing effects on fine root NPP did not show a clear trend across the climate gradient. We expected negative effects of grazing on root biomass and NPP in our dry sites as seen for aboveground plant biomass in drylands of Brazil (Manhães et al. 2022), Australia (Eldridge et al. 2016), and North America (Bestelmeyer et al. 2018; Jordan et al. 2022). However, our study did not manipulate levels of grazing across sites, which limits direct comparisons. We observed significant increases in fine root NPP under cattle grazing in our driest grassland (CPER), consistent with previous studies showing that grazing maintains the dominance of *B. gracilis* , a grazing‐tolerant C4 perennial grass with high root: shoot ratios and fine root production rates relative to species that are dominant in the absence of grazing (Derner et al. 2006; Milchunas 2009). The evolutionary context of dominant plant traits matters, which may strongly mediate outcomes of disturbance or its exclusion on productivity. For example, grazing at Kruger in combination with low‐intensity and frequent fire may promote short‐statured, shallow‐rooted lawn grasses, which are characterized by rapid regrowth and high NPP due to lower structural investment, over bunch grasses (Hempson et al. 2015). In another evolutionary feedback, mixed feeders and browsers also maintain the openness of grazing lawns which confers predator avoidance (Voysey et al. 2021). Alternatively, grazing may shift diversity of grasses via removal of dominant, palatable species and facilitate community shifts to larger, unpalatable species that are also fire‐resistant in taller forms (Archibald et al. 2019; Koerner et al. 2018). The differential outcome of plant life‐history strategies and subsequent effects on BNPP in response to grazing is thus important within the context of evolutionary history with fire (Archibald et al. 2019) and may be independent of productivity or precipitation (Koerner et al. 2018).

### 4.2 Other Physiological, Edaphic, and Abiotic Factors That Underly BNPP

In addition to long‐term shifts in vegetation structure and traits, short‐term physiological processes may also influence responses to herbivory. For example, we observed differences in fine root NPP between two sites with bison grazing (KPBS and CDR), where fine root NPP decreased at KPBS and increased at CDR with herbivory. Although similar in mean annual precipitation and plant community composition, KPBS and CDR differed in the duration of herbivory manipulations. The grazing manipulation at CDR was conducted for four years (compared to > 30 years at KPBS), and because we sampled post‐experiment, we may be capturing compensatory growth after grazing ceased (Oesterheld and McNaughton 1991). BNPP in grazed CDR plots disproportionately suppressed what would have been a negative linear effect of grazing on BNPP with water availability (Figure 4c, dark points; Supplemental Material). Future work should explicitly separate short‐term compensatory responses from longer‐term shifts in community composition.

Shifts in allocation of biomass belowground may further explain variability in fine root NPP, but allocation was not consistently shaped by changes in disturbance regime in our study. Allocation theory states that along resource–climate gradients, shifts in the most limiting resource drive changes in above‐ versus belowground allocation (Aerts et al. 1991; Grime 1973; Tilman 1985), and the resulting optimal partitioning maximizes total productivity (Poorter et al. 2012; Qi et al. 2019). However, such responses may be highly context dependent (Farrior et al. 2013). Grazing in dry sites appeared to result in a higher root mass fraction if plants allocate more resources belowground to quickly regrow throughout a growing season when resources are available. Otherwise, there were no consistent patterns in root mass fraction across sites or according to disturbance types within a site that might explain generalizable changes to fine root NPP. Rather than considering biomass allocation ratios, the available root stock, therefore, may underly shifts in productivity. Fine root stocks only positively correlated with fine root NPP in the two highest disturbance treatments (frequent fire and herbivory). These patterns, however, were influenced by one of our humid sites (ABS), which had notably higher fine root stocks attributed to palmetto roots ( *S. repens* and *S. etonia* ), rather than herbaceous species. Palmettos are a notably slow‐growing, “woody” monocot that lack true wood with relatively large above– and belowground biomass stock in this system (Abrahamson and Abrahamson 2009). Therefore, once again, understanding the site‐specific, ecological context is critical for disentangling the relationship between above and belowground dynamics.

Edaphic factors also matter for root dynamics. Clay content, for example, can strongly impact root growth and elongation (Gerard et al. 1982). In the bison‐grazed sites, soil type also differed and likely explains divergence in BNPP responses to herbivory; the soils at KPBS, with 37% clay content, may impose potentially different physical and nutritional constraints (Johnson et al. 2014) on fine root NPP compared to the sandy CDR site. In African savannas, establishment of dominant plant functional groups similarly depends on parent material, soil texture, and nutrient availability, in addition to herbivore functional guilds (Hempson et al. 2015); At Kruger, stoloniferous lawns often forming in granitic landscapes with higher water availability (SKU, PKOP) and bunch grasses generally forming in basalt landscapes (SAT) in response to grazing pressure (but see Koerner et al. 2018). Thus, our results likely reflect multiple interacting mechanisms that we did not fully disentangle.

Soil depth and rooting profiles beyond 20 cm were not considered in this study but also are important to consider. We expect the highest concentration and shift in fine roots to occur in the top 20 cm even in the mesic sites (c.f., Kitchen et al. 2009), but our choice to consider BNPP in the top 20 cm may mask deep root dynamics attributed to woody plants or deep‐rooting grasses like *B. gracilis* (present at CPER). For example, (Pellegrini et al. 2020) found that fine root biomass in our most mesic site (CDR) was concentrated in the top 20 cm but frequent burning resulted in greater losses of coarse roots up to 40 cm. While our study may underestimate fire effects on coarse roots, we chose to focus on fine roots especially as key components for carbon cycling in savannas.

In addition to site‐level plant physiology or soil properties driving variability in fine root NPP responses, nuances specific to disturbance types within site are also likely at play. Our one‐way experimental design does not allow us to necessarily compare the effects of fire to herbivory directly, however. Nevertheless, fire and herbivory may functionally differ in timing and duration of biomass removal. Managers of our study sites conduct prescribed burns outside of the peak growing season (except ABS, which burns year‐round). The prescribed burns are surface fires, during which fire is largely non‐selective and consumes available aboveground biomass, particularly short‐statured plants and litter. In contrast, herbivore consumption is temporally variable depending on plant phenology, season and forage availability, and/or land management. In addition, the destruction and removal of woody plants by browsers and mixed feeders in Kruger represents a mechanism of woody plant reduction that is not present at comparable densities at the other sites and is not necessarily limited to shorter woody plants. Consistent with this, we observed a decrease in woody aboveground biomass in fire suppression plots at two Kruger sites (SAT, SKU) open to large herbivores compared to plots where both fire and herbivores are excluded. Thus, herbivore manipulation of woody plants, regardless of climate, also likely reflects the effects of different herbivore guilds (e.g., browsers and mixed feeders versus grazers) across sites.

### 4.3 Implications for C Storage Under Global Change

Our results ultimately present important considerations for ecosystem C storage under shifting disturbance regimes in grasslands and savannas, globally. While aboveground biomass increases in the absence of disturbance (Higgins et al. 2007; Reich et al. 2001) imply gains in ecosystem C storage, our results do not always indicate synergistic or parallel gains belowground. In grasslands and savannas, a fuller picture that includes belowground stocks challenges the benefit of allowing woody thickening or afforestation since the majority of carbon is stored in roots and soil. Even though gains in fine root stocks were sometimes synchronous with gains in woody biomass for our study, loss of the herbaceous layer specifically has impactful consequences for C sequestration and storage (Bai and Cotrufo 2022) because grasses maintain a large contribution to the soil C pool despite increasing woody cover (Coetsee et al. 2023; Wigley et al. 2020; Zhou et al. 2023). Our results suggest that swings in ecosystem C storage may be more pronounced in wet savannas with shifting disturbance regimes, whereas grasslands and drier savannas exhibit more resilience.

Although we did not explicitly address climate change in this study, grasslands and savannas are also experiencing increased rainfall variability and droughts of novel duration and magnitude (Calvin et al. 2023) that, in tandem with changes to disturbance regimes, will also affect productivity rates and global C cycling. Given the greater relative magnitude of BNPP responses we observed in wetter savannas, coupled fire–climate effects on productivity may therefore be amplified in these systems, increasing variability in soil C storage. Conversely, on the other end of the spectrum, the hallmark of semi‐arid grasslands is their ability to respond quickly to precipitation pulse events (Ogle and Reynolds 2004), and carbon‐related responses are more coupled to water‐related pulses in drier biomes (Reich et al. 2025). Thus, future variability in C cycles in the driest grasslands may be more tied to precipitation extremes rather than disturbance if fuel loads are tipped below thresholds for burning. In addition, climate‐driven shifts in herbivore behavior, such as increased woody plant use by African savanna mixed feeders during drought (Abraham et al. 2019), may further alter vegetation structure and reduce belowground C allocation by removing both herbaceous contributions belowground via drought and woody contributions via increased herbivory. Our findings collectively show that belowground processes in grassland and savanna ecosystems are fundamentally dependent on disturbance regimes, aridity and their interactions, with profound implications for global C cycling and storage under global change.

## Author Contributions

**Courtney M. Currier:** conceptualization, methodology, investigation, writing – original draft, validation, visualization, writing – review and editing, formal analysis, project administration, data curation, supervision, software. **Christian Parrinello:** investigation, writing – review and editing, supervision, project administration, data curation. **Charlotte Dick:** investigation, writing – review and editing, data curation. **David J. Augustine:** writing – review and editing, validation, resources, investigation. **John M. Blair:** investigation, writing – review and editing, resources. **Elizabeth H. Boughton:** investigation, writing – review and editing, resources. **Corli Coetsee:** investigation, writing – review and editing, resources. **Aaron S. David:** investigation, writing – review and editing, resources. **Justin D. Derner:** investigation, writing – review and editing, resources. **Sarah E. Hobbie:** investigation, writing – review and editing, resources. **Forest Isbell:** investigation, writing – review and editing, resources. **Kamogelo S. Modimola:** investigation, writing – review and editing, project administration, supervision. **Peter B. Reich:** investigation, validation, writing – review and editing, resources. **Benjamin Wigley:** investigation, writing – review and editing, resources. **Kally Worm:** investigation, project administration, resources, writing – review and editing, supervision. **Lydia H. Zeglin:** investigation, writing – review and editing, resources. **Adam Pellegrini:** funding acquisition, conceptualization, investigation, writing – original draft, methodology, validation, writing – review and editing, project administration, supervision, resources, data curation, visualization.

## Funding

This work was supported by UK Research and Innovation (Grant EP/X042863/1), South African National Parks (Grant SP‐GK‐1399), National Science Foundation (Grants DEB‐0620652, DEB‐1234162, DEB‐1831944, DEB 2425352, DBI‐2021898, and DEB‐2025849), Agricultural Research Service and Long Term Agroecosystem Research Network.

## Conflicts of Interest

The authors declare no conflicts of interest.

## Supporting information


**Figure S1:** Geographic distribution of study sites. (a) Global map showing study site locations relative to global aridity patterns; (b) Sites located across North America; (c) Sites located in Kruger National Park, South Africa; (d) Long‐term climate data computed from Zomer et al. (2022), over the 1970–2000 time period, and study year rainfall amount (mm).
**Table S1:** Description of fire and herbivore manipulations.
**Figure S2:** Belowground relationships of fine root turnover (year^−1^) with climate (aridity index). Large points represent site‐level means, regardless of disturbance treatment, ± standard error. Light points represent raw values, and solid lines indicate significant predicted fits back‐transformed from generalized mixed effects models using Tweedie distribution and log‐link, with plot nested within site as the random effect for response variables. Dotted lines represent 95% confidence intervals around predicted fit. *N* = 770 root cores, (385 standing stock and 385 in‐growth). Statistical output is reported in Table S2.
**Figure S3:** Aboveground relationships of (a) woody stem density (stems m^−2^), (b) herbaceous aboveground stocks (g m^−2^), and (c) herbaceous aboveground stocks versus stem density, regardless of disturbance. Large points represent site‐level means, regardless of disturbance treatment, ± standard error. Light points represent raw values, and solid lines indicate significant predicted fits back‐transformed from generalized mixed effects models using Tweedie distribution and log‐link, with plot nested within site as the random effect for response variables. Dotted lines represent 95% confidence intervals around predicted fit. Statistical output are in Table S2.
**Figure S4:** Aboveground relationships of woody stem density (stems m^−2^), herbaceous aboveground stocks (g m^−2^), and herbaceous aboveground stocks versus stem density under fire suppression (a–c) and herbivore exclusion (d–f). Large points represent site‐level means ± standard error. Light points represent raw values, and solid lines indicate significant predicted fits back‐transformed from generalized mixed effects models using Tweedie distribution and log‐link, with plot nested within site as the random effect for response variables. Dotted lines represent 95% confidence intervals around predicted fit. Statistical Output are in Table S2.
**Figure S5:** Belowground relationships of fine root NPP (g m^−2^ year^−1^) versus fine root stock (g m^−2^). Large points represent site‐level means, regardless of disturbance treatment, ± standard error. Light points represent raw values, and solid lines indicate significant predicted fits back‐transformed from generalized mixed effects models using Tweedie distribution and log‐link. Dotted lines represent 95% confidence intervals around predicted fit. Statistical Output are in Table S2.
**Figure S6:** Absolute change in fine root mass fraction across and within study sites for (a) fire treatments and (b) herbivore treatments. Bars represent means ± standard error. Site legend (in order from most arid to least arid): Central Plains Experimental Range (CPER), Satara (SAT), Skukuza (SKU), Pretoriuskop (PKOP), Konza Prairie Biological Station (KPBS), Archbold Biological Station (ABS), Cedar Creek LTER (CDR).
**Table S2:** Aridity and vegetation relationships above and belowground, not parsed by disturbance.
**Table S3:** Fine versus coarse root stocks (g m^−2^) and net primary productivity (g m^−2^ year^−1^) across sites and disturbance treatments.
**Table S4:** Climate and disturbance effects on relative and RII fine root responses.
**Table S5:** Above and belowground predictors of fine root NPP across disturbance treatments.
**Table S6:** Contribution to gross and relative change in net biomass stock due to fine root stocks.

## Data Availability

The data and code supporting the results are available on Dryad, https://doi.org/10.5061/dryad.zw3r228q5.
