# Supplementary material for: Belowground Productivity in Grasslands and Savannas Depends on Shifting Fire and Herbivory Regimes
Source: Glob Chang Biol. 2026 Nov 13;32(11):e71202. doi: 10.1111/gcb.71202 (PMC13763163; doi:10.1111/gcb.71202)
Supplement: Supplementary file 1 [file GCB-32-e71202-s001.docx]

**Supplementary Material for: Belowground productivity in grasslands and savannas depends on shifting fire and herbivory regimes**

Prepared for submission to *Global Change Biology* by

Courtney M. Currier ^1,2*^, Christian Parrinello ^1^, Charlotte Dick ^1^, David J. Augustine ^3^, John M. Blair ^4^, Elizabeth H. Boughton ^5^, Corli Coetsee ^6,7^, Aaron S. David ^8^, Justin D. Derner ^3^, Sarah E. Hobbie ^9^, Forest Isbell ^9^, Kamogelo S. Modimola ^10^, Peter B. Reich ^11,12^, Benjamin Wigley ^6,7,13^, Kally Worm ^9^, Lydia H. Zeglin ^4^, and Adam Pellegrini ^1,2*^

^1^ Department of Plant Sciences, University of Cambridge, CB2 3EA, United Kingdom

^2^ Department of Earth System Science, Doerr School of Sustainability, Stanford University, Stanford, CA 94305, USA

^3^ USDA-Agricultural Research Service, Center for Agricultural Resources Research, Rangeland Resources & Systems Research Unit, Fort Collins, CO 80526, USA

^4^ Division of Biology, Kansas State University, Manhattan, KS 66506, USA

^5^ Archbold Biological Station and Buck Island Ranch, Lake Placid, FL 33852, USA

^6^ Savanna Research Node, Scientific Services, SANParks, Skukuza, 1350, South Africa

^7^ School of Natural Resource Management, Nelson Mandela University, George Campus, 6530, South Africa

^8^ Archbold Biological Station, Venus, FL 33960, USA

^9^ Department of Ecology, Evolution and Behavior, University of Minnesota, St. Paul, MN 55108, USA

^10^ Environmental & Geographical Science, University of Cape Town, Rondebosch, 7701, South Africa

^11^ Department of Forest Resources, University of Minnesota, St. Paul, MN 55108, USA

^12^ Institute for Global Change Biology and School for the Environment and Sustainability, University of Michigan, Ann Arbor, MI 48109, USA

^13^ Plant Ecology, University of Bayreuth, Universitaetsstrasse 30, 95447 Bayreuth, Germany

***Corresponding Authors** can be reached at [ccurrier@stanford.edu](mailto:ccurrier@stanford.edu) and [afapelle@stanford.edu](mailto:afapelle@stanford.edu)

**Detailed study site descriptions in order of aridity index**

*Colorado Plains Experimental Range (CPER)*

The Colorado Plains Experimental Range (CPER), located in north-central CO, USA, represents a semi-arid, shortgrass steppe. Mean annual precipitation is approximately 341 mm. Grazing by bison and, more recently, cattle have been a dominant influence, whereas fires were historically limited to dry seasons that followed unusually wet years, with an estimated return interval >10 years (Guyette *et al.*, 2012). Aridisols and Entisols formed in a mosaic of alluvium and eolian sediments constitute the soil types across the range; our sampling focused on fine sandy loam regions of eolian and alluvium deposits over residuum weathered from sandstone (Augustine *et al.*, 2024). Vegetation is dominated by the C4 perennial blue grama (*Bouteloua gracilis*). Long-term grazing exclusion over seven decades shifts grass dominance from *B. gracilis* to the C3 perennial mid-grass, *Pascopyrum smithii* (Augustine *et al.*, 2017). Sub-shrubs (e.g., *Artemisia* spp.) and prickly pear (*Opuntia* spp.) may establish, but woody cover is minimal compared to our other study sites. A fire manipulation, with plots 0.04 ha in size, took place from 2007–2016 in a pasture where cattle were excluded (Dufek *et al.*, 2018). Fire frequencies during the manipulation study were: unburned, triennial burn (spring and fall), and annual burn (spring and fall). In addition, long-term cattle grazing exclosures, 0.5–1 ha in size, were established at CPER in 1939 within pastures grazed since that time by cattle at either moderate or heavy stocking rates, highlighting the site’s extensive history of grazing research. We grouped seasonal treatments together according to burn frequency and grazing intensity treatments together according to presence or absence of cattle in the final analyses; seasonal timing of fire and grazing intensity did not have robust effects across our response variables for this study.

*Kruger National Park: Satara (SAT), Skukuza (SKU), Pretoriuskop (PKOP)*

Our sites in Kruger National Park, South Africa, span a gradient of climate and soil conditions. Historically, fires occured at intervals of approximately four years across the park (Van Wilgen, 2000). Kruger supports a highly diverse assemblage of large herbivores, including grazers such as wildebeest (*Connochaetes taurinus*) and zebra (*Equus quagga*), browsers such as giraffe (*Giraffa camelopardalis*) and kudu (*Tragelaphus strepsiceros*), and mixed feeders such as impala (*Aepyceros melampus*) and elephant (*Loxodonta africana*). Experimental Burn Plots, 7 ha in size, were established at all three sites in 1956 and continue to the present and are open to all large herbivores. We sampled plots that are unburned as well as burned triennially and annually in spring.

The driest site, Satara (SAT), has a mean annual precipitation of 547 mm. Soils are mainly moderately deep Shortlands–Swartland soil that are red structured clays situated on the Sabie River Basalt Formation with dolerite dykes (Venter & Govender, 2012). Grass cover is extensive here, dominated by C_4_ species like *Bothriochloa radicans*, *Panicum coloratum*, and *Digitaria eriantha*. The canopy, dominated by *Combretum* spp. and *Senegalia nigrescens*, is relatively open even under fire suppression compared to the other two Kruger sites. The herbivore exclusion plot, 900 ha in size, was established adjacent to one replicate fire block near the Satara rest camp in 2005 by University of Cape Town (UCT) (Wigley-Coetsee *et al.*, 2022) and is not prescriptively burned.

Skukuza (SKU) was our intermediate rainfall site at Kruger with a mean annual precipitation of 553 mm. Skukuza soils are well-drained sandy loams, derived from granitic parent material ranging from the Glovelly–Hutton–Glenrosa (Skukuza string), Fernwood–Kroonstad and Hutton–Glovelly–Fernwood (Napi string), and Glenrosa-Hutton forms (Nwaswitshaka string) (Venter & Govender, 2012). The grass community is dominated by C4 grasses, comprising *Digitaria eriantha, Setaria sphacelata* and *Megathyrsus maximus* (Smith *et al.*, 2013). The dominant tree species here are an assemblage of *Combretum* spp. and *Terminalia sericea*. The N’Kuhlu herbivore exclusion plot, 139 ha in size, was established in 2002 on the Sabie River approximately 24 km away from the Skukuza rest camp (Wigley-Coetsee *et al.*, 2022), but situated on a similar soil type with similar rainfall amount as the SKU experimental burn plots. Like the UCT Satara herbivore exclosure, N’Kuhlu is not prescriptively burned.

Pretoriuskop (PKOP) represents the wettest site at Kruger, with a mean annual precipitation of 746 mm. Soils at PKOP are well-drained and sandy loam, derived from granitic parent material primarily of the deep red sand formations (Venter & Govender, 2012). The grass community is dominated by the C_4_ grasses, comprising *Hyperthelia dissoluta, Elionurus argenteus* and *Megathyrsus maximus*. *Terminalia sericea* dominates the tree community at PKOP. We did not sample a grazing exclosure at PKOP, and it is the only site of seven for this study without comparisons for herbivory effects.

*Konza Prairie Biological Station (KPBS)*

Our next wettest site was a mesic tallgrass prairie, Konza Prairie Biological Station (KPBS), located in Kansas, USA. KPBS is located in the Flint Hills ecoregion, where a historic fire return interval of 3–4 years via wildfire and indigenous burning along with bison grazing maintained the largest extent of native tallgrass prairie in the United States (Reichman, 1987). KPBS has a mean annual precipitation of 834 mm. The watersheds of KPBS have a distinct topography, where underlying soil is very heterogeneous between upland and lowland topographic positions. We sampled from lowland areas within each watershed on silty clayey loam soils of colluvium derived from chert over residuum weathered from limestone, from the Tully complex soil map units (Connell *et al.*, 2020). Dominant grasses are perennial C4 species, including *Andropogon gerardii*, *Schizachyrium scoparium*, *Sorghastrum nutans*, and *Panicum virgatum*. Dominant woody species include *Cornus drumondii*, *Rhus* spp., and *Prunus americana*. The ungrazed fire experiments at KPBS commenced between 1972 and 1979 in replicated watershed units, on average ~66 ha in size; we selected units that have not been burned for 20 years, as well as units that are burned every 4 and 2 years. The historically abundant native grazer, bison, was re-introduced to KPBS between 1979 and 1988, creating a fire by herbivore factorial design across the entire site; we paired our ungrazed, biennially burned plots with two biennially burned units grazed by bison.

*Archbold Biological Station (ABS)*

Archbold Biological Station (ABS) is located in Florida, USA, and is representative of the flatwoods and scrubby flatwoods ecosystem native to the southeastern USA (Abrahamson *et al.*, 1984). This ecosystem has a long evolutionary history of frequent, lightning-ignited fire, with a historic fire return interval of 6–20 years for flatwoods and scrubby flatwoods (Noss, 2018). South Florida slash pine (*Pinus densa*) canopies are relatively open with an understory dominated by both herbaceous species and shrubs. Dominant grass species include wiregrass (*Aristida beyrichiana*) and cutthroat grass (*Coleataenia abscissa*). Dominant shrubs include several species of oaks (*Quercus inopina*, *Quercus chapmanii, Quercus myrtifolia, Quercus geminat*a), heaths (*Lyonia fruticose*, *Lyonia lucida*, *Bejaria racemosa*), and palmettos (*Sabal etonia*, *Serenoa repens*). Mean annual precipitation is 1,249 mm, but high potential evapotranspiration makes this site slightly less humid compared to Cedar Creek (below). Soils are sands derived almost exclusively from quartzite of the Miocene Hawthorn formation, with a deep spodic layer occurring down to a meter in some areas (Abrahamson *et al.*, 1984). Regular prescribed burning of replicated, ungrazed pastures, 4–72 ha in size, commenced in 1977 and takes place during the growing season, but may extend year-round. We selected units that are burned at 20–60 year, 6–9 year, and 1–5 year frequencies. Cattle grazing was introduced to the region in 1970’s, and the conversion of natural land involved addition of exotic pasture grasses and shrub removal; the cattle grazed pasture, which is burned every 1–3 years, used in this study was acquired and managed by ABS in 2002.

*Cedar Creek Long Term Ecological Research Site (CDR)*

Cedar Creek Long Term Ecological Research site (CDR) is our final site, located in Minnesota, USA. Representing a temperate broadleaf savanna, the historic fire return interval for CDR was 1–3 years prior to 1920, after which fire was excluded for approximately 40 years. The mean annual precipitation is 735 mm. Soils are fine to medium sands and well drained on parent material dominated by quartzite of the Sartell and Zimmerman soil series (Grigal, 1974). The grasses comprise a mix of C3 and C4 species, with C4 being more abundant and including *Andropogon gerardii*, *Schizachyrium scoparium*, and *Sorghastrum nutans*. The woody plant community is dominated by oaks, namely *Quercus macrocarpa* and *Q. ellisoidalis*, as well as shrubs like *Corylus* spp. The fire manipulation experiment was established in 1964 in burn units, 3–30 ha in size, and cattle were excluded. We selected unburned units as well as 0.3–0.5 fires year^-1^ and 0.7–0.8 fires year^-1^ spring burn frequencies. Bison were introduced to several burn unit enclosures from 2018–2023; we sampled from a bison enclosure with a 0.5 fires year^-1^ burn frequency.

**Fig. S1 | Geographic distribution of study sites.** a) Global map showing study site locations relative to global aridity patterns; b) Sites located across North America; c) Sites located in Kruger National Park, South Africa; d) Long-term climate data computed from (Zomer *et al.*, 2022), over the 1970‒2000 time period, and study year rainfall amount (mm).

**Table S1 | Description of fire and herbivore manipulations.**

**Supplemental Results**

*Trends in woody and herbaceous biomass across sites*

First, woody cover, represented by total stem density (individuals m^-2^), was greater in wetter sites; there was a rise in stem density with water availability, from being mostly absent in the driest site to 9.42 ± 4.44 stems m^-2^ (means ± standard error, inclusive of all disturbance treatments) in the wettest site (Fig. S3a; Table S2). This approximately exponential increase differed from the quadratic relationship between herbaceous biomass and water availability (Fig. S3b; Table S2), resulting in a relationship where the gains in stem density in wet sites corresponded with a decline in herbaceous aboveground biomass (Fig. S3c; Table S2). This points to a transition from herbaceous biomass being limited by water to being limited by other factors, namely light, with woody thickening.

We used the disturbance treatments to understand whether the exclusion of fire and large herbivores decreased herbaceous biomass more in wetter climates. Indeed, we found that woody stem density increased under fire exclusion (Fig. S4a; Table S2) at wetter sites; herbaceous biomass declined with fire and grazer exclusion to a greater extent in the wet sites (Fig. S4b,e; Table S2), which generally also experienced the greatest gains in stem density (Fig. S4c,f; Table S2). We did not find significant increases in woody biomass across the aridity gradient under herbivore exclusion (Fig. S4d; Table S2). However, that said, when considering herbivore manipulation plots in which woody plants were present and met measurement criteria, we observed significant relative increases in woody stem density and biomass in plots open to herbivores compared to exclosures (Fig. 6, main text; Table S6). The exception was SAT, which showed a decrease in relative woody biomass with herbivory but an increase in overall stem density, suggesting a loss of larger woody individuals in the presence of browsers.

*Sensitivity analyses: Are drivers of BNPP relationships attributed to single influential sites?*

To evaluate whether results were disproportionately influenced by any site, we conducted sensitivity analyses for all aridity-BNPP models (Fig. 3, main text), BNPP vs. aboveground biomass models (Fig. 4, main text), and BNPP vs. belowground biomass models (Fig. 5, main text) by re-fitting each model with one site excluded at a time and comparing new estimates to the full-data model. For Fig. 4, we also checked both effects of CDR on scaling of the stem density explanatory variable and the overall model effect potentially driven by CDR having high stem density. Model fits inclusive of all sites are in Table S4.

For BNPP–aridity relationships (Fig. 3, main text), the significant relationship in the frequent fire model remained significant after excluding CDR (β = 1.32, p < 0.01, R^2^m = 0.10, R^2^c = 0.15). In the grazing mode, excluding CDR revealed a significant linear aridity effect (β = -1.90, p = 0.027, R^2^m = 0.13, R^2^c = 0.31) that was not detected in the full model, while excluding CPER eliminated the significant quadratic aridity effect (β = 1.36, p = 0.201, R^2^m = 0.09, R^2^c = 0.51). The aridity–BNPP relationship in the fire suppression model was non-significant regardless of site exclusion.

For BNPP–stem density relationships (Fig. 4, main text), the positive trend observed in grazed plots was dependent on CDR; excluding CDR's data rendered the relationship non-significant (β = 0.020, p = 0.863, R^2^m = 0.005, R^2^c = 0.42), reflecting CDR's substantially higher stem density relative to other sites due to the presence of densely re-sprouting oak stems. No relationship between stem density and BNPP was detected in ungrazed plots regardless of site exclusion.

For BNPP–fine root stock relationships (Fig. 5, main text), ABS was the most influential site across treatments. In frequent-fire plots, the relationship was marginally significant in the full model but lost significance when ABS was excluded (β = 0.553, p = 0.118, R^2^m = 0.034, R^2^c = 0.62), indicating ABS contributed to the overall positive effect of root stocks on fine root NPP under frequent fire. In grazed plots, the significant positive relationship strengthened when ABS was excluded (β = 1.44, p = 0.0026, R^2^m = 0.14, R^2^c = 0.67), indicating ABS weakened the BNPP–fine root stock relationship. Relationships in suppressed fire, intermediate-fire treatments, and ungrazed plots, were not significant regardless of site exclusion.

**Supplemental Figures**

**Fig. S2 | Belowground relationships of fine root turnover (yr^-1^) with climate (aridity index).** Large points represent site-level means, regardless of disturbance treatment, ± standard error. Light points represent raw values, and solid lines indicate significant predicted fits back-transformed from generalized mixed effects models using Tweedie distribution and log-link, with plot nested within site as the random effect for response variables. Dotted lines represent 95% confidence intervals around predicted fit. N = 770 root cores, (385 standing stock and 385 in-growth). Statistical output is reported in Table S2.

**Fig. S3 | Aboveground relationships of a) woody stem density (stems m^-2^), b) herbaceous aboveground stocks (g m^-2^), and c) herbaceous aboveground stocks vs. stem density, regardless of disturbance.** Large points represent site-level means, regardless of disturbance treatment, ± standard error. Light points represent raw values, and solid lines indicate significant predicted fits back-transformed from generalized mixed effects models using Tweedie distribution and log-link, with plot nested within site as the random effect for response variables. Dotted lines represent 95% confidence intervals around predicted fit. Statistical output are in Table S2.

**Fig. S4 | Aboveground relationships of woody stem density (stems m^-2^), herbaceous aboveground stocks (g m^-2^), and herbaceous aboveground stocks vs. stem density under fire suppression (a–c) and herbivore exclusion (d–f).** Large points represent site-level means ± standard error. Light points represent raw values, and solid lines indicate significant predicted fits back-transformed from generalized mixed effects models using Tweedie distribution and log-link, with plot nested within site as the random effect for response variables. Dotted lines represent 95% confidence intervals around predicted fit. Statistical Output are in Table S2.

**Fig. S5 | Belowground relationships of fine root NPP (g m^-2^ yr^-1^) vs. fine root stock (g m^-2^).** Large points represent site-level means, regardless of disturbance treatment, ± standard error. Light points represent raw values, and solid lines indicate significant predicted fits back-transformed from generalized mixed effects models using Tweedie distribution and log-link. Dotted lines represent 95% confidence intervals around predicted fit. Statistical Output are in Table S2.

**Fig. S6 | Absolute change in** **fine root mass fraction across and within study sites for a) fire treatments and b) herbivore treatments.** Bars represent means ± standard error. Site legend (in order from most arid to least arid): Central Plains Experimental Range (CPER), Satara (SAT), Skukuza (SKU), Pretoriuskop (PKOP), Konza Prairie Biological Station (KPBS), Archbold Biological Station (ABS), Cedar Creek LTER (CDR).

**Supplemental Tables**

**Table S2 | Aridity and vegetation relationships above and belowground, not parsed by disturbance**

**Table S3 | Fine versus coarse root stocks (g m^-2^) and net primary productivity (g m^-2^ yr^-1^) across sites and disturbance treatments.**

**Table S4 | Climate and disturbance effects on relative and RII fine root responses.**

**Table S5 | Above and belowground predictors of fine root NPP across disturbance treatments.**

**Table S6** **| Contribution to gross and relative change in net biomass stock due to fine root stocks.**

**References**

Abrahamson WG, Johnson AF, Layne JN, Peroni PA. 1984. Vegetation of the Archbold Biological Station, Florida: An Example of the Southern Lake Wales Ridge. *Florida Scientist* 47: 209–250.

Augustine DJ, Derner JD, Milchunas D, Blumenthal D, Porensky LM. 2017. Grazing moderates increases in C3 grass abundance over seven decades across a soil texture gradient in shortgrass steppe. *Journal of Vegetation Science* 28: 562–572.

Augustine DJ, Derner JD, Porensky LM, Hoover DL, Ritten JP, Kearney SP, Ma L, Peck D, Wilmer H, Group the CS. 2024. The LTAR Grazing Land Common Experiment at the Central Plains Experimental Range: Collaborative adaptive rangeland management. *Journal of Environmental Quality* 53: 904–912.

Connell RK, Nippert JB, Blair JM. 2020. Three Decades of Divergent Land Use and Plant Community Change Alters Soil C and N Content in Tallgrass Prairie. *Journal of Geophysical Research: Biogeosciences* 125: e2020JG005723.

Dufek NA, Augustine DJ, Blumenthal DM, Kray JA, Derner JD. 2018. Dormant-Season Fire Inhibits Sixweeks Fescue and Enhances Forage Production in Shortgrass Steppe. *Fire Ecology* 14: 33–49.

Grigal DF. 1974. *Soils of the Cedar Creek Natural History Area*. St. Paul: Agricultural Experiment Station, University of Minnesota.

Guyette RP, Stambaugh MC, Dey DC, Muzika R-M. 2012. Predicting Fire Frequency with Chemistry and Climate. *Ecosystems* 15: 322–335.

Noss RF. 2018. *Fire Ecology of Florida and the Southeastern Coastal Plain*. University Press of Florida.

Reichman OJ. 1987. *Konza Prairie: A Tallgrass Natural History*. University Press of Kansas.

Smith MD, van Wilgen BW, Burns CE, Govender N, Potgieter ALF, Andelman S, Biggs HC, Botha J, Trollope WSW. 2013. Long-term effects of fire frequency and season on herbaceous vegetation in savannas of the Kruger National Park, South Africa. *Journal of Plant Ecology* 6: 71–83.

Van Wilgen B Biggs, HC**, O’Regan, SP***, Mare, N**. 2000. A fire history of the savanna ecosystems in the Kruger National Park, South Africa, between 1941 and 1996. *South African Journal of Science* 96: 167–178.

Venter FJ, Govender N. 2012. A geomorphic and soil description of the long-term fire experiment in the Kruger National Park, South Africa. *Koedoe* 54: 10 pages.

Wigley-Coetsee C, Strydom T, Govender D, Thompson DI, Govender N, Botha J, Simms C, Manganyi A, Kruger L, Venter J, *et al.* 2022. Reflecting on research produced after more than 60 years of exclosures in the Kruger National Park. *Koedoe* 64.

Zomer RJ, Xu J, Trabucco A. 2022. Version 3 of the Global Aridity Index and Potential Evapotranspiration Database. *Scientific Data* 9: 409.
